# Probiotics as modulators of gut-brain axis for cognitive development

**DOI:** 10.3389/fphar.2024.1348297

**Published:** 2024-02-20

**Authors:** Akash Kumar, Bhagavathi Sundaram Sivamaruthi, Swarnima Dey, Yogesh Kumar, Rishabha Malviya, Bhupendra G. Prajapati, Chaiyavat Chaiyasut

**Affiliations:** ^1^ Department of Food Technology, SRM University, Sonipat, Delhi, India; ^2^ Office of Research Administration, Chiang Mai University, Chiang Mai, Thailand; ^3^ Innovation Center for Holistic Health, Nutraceuticals, and Cosmeceuticals, Faculty of Pharmacy, Chiang Mai University, Chiang Mai, Thailand; ^4^ Amity Institute of Food Technology, Amity University, Noida, Uttar Pradesh, India; ^5^ Department of Paramedical and Allied Sciences, School of Medical and Allied Sciences, Galgotias University, Greater Noida, Uttar Pradesh, India; ^6^ Shree S. K. Patel College of Pharmaceutical Education and Research, Ganpat University, Mehsana, India

**Keywords:** cognition, gut-brain axis, *Lactobacillus*, *Bifidobacterium*, inflammation

## Abstract

Various microbial communities reside in the gastrointestinal tract of humans and play an important role in immunity, digestion, drug metabolism, intestinal integrity, and protection from pathogens. Recent studies have revealed that the gut microbiota (GM) is involved in communication with the brain, through a bidirectional communication network known as the gut-brain axis. This communication involves humoral, immunological, endocrine, and neural pathways. Gut dysbiosis negatively impacts these communication pathways, leading to neurological complications and cognitive deficits. Both pre-clinical and clinical studies have demonstrated that probiotics can restore healthy GM, reduce intestinal pH, and reduce inflammation and pathogenic microbes in the gut. Additionally, probiotics improve cell-to-cell signaling and increase blood-brain-derived neurotrophic factors. Probiotics emerge as a potential approach for preventing and managing neurological complications and cognitive deficits. Despite these promising findings, the safety concerns and possible risks of probiotic usage must be closely monitored and addressed. This review article provides a brief overview of the role and significance of probiotics in cognitive health.

## 1 Introduction

Cognition was once assumed to be controlled only by the central nervous system (CNS) (Zhu et al., 2020), but recent studies have revealed that a diverse range of factors plays a role in its regulation and influence. The gastrointestinal tract of humans is a habitat of various microbial communities, referred to as gut microbiota (GM) (Patel et al., 2023). GM may impact host health and is regulated by various factors such as stress, age, food, and host genetics (Long-Smith et al., 2020). The gut and brain communicate through a bidirectional communication network, known as the gut-brain axis (GBA) (Kumar et al., 2023). GM dysbiosis may alter brain structure, brain vascular physiology, and blood-brain barrier (BBB) permeability which may cause neurological disorders and cognitive impairment (Zhu et al., 2020). Therefore, GM may be a potential target for preventing and treating cognitive impairment (Sun et al., 2020; Eastwood et al., 2021).

Probiotics are “live microorganisms that, when administrated in sufficient amounts, confer a health benefit to the host” (Martín and Langella_2019). Probiotic administration results in the restoration of GM, changes in microbiota-derived metabolites, reduction in inflammation, and maintains hypothalamic pituitary adrenal (HPA) axis function and gut barrier integrity (Plaza-Diaz et al., 2019). In recent years, pre-clinical and clinical studies have described the role of probiotics in cognitive health (Sivamaruthi et al., 2019; Thangaleela et al., 2022). This review aims to provide a concise summary of the probiotic potential for preventing and treating cognitive impairments.

## 2 Gut-brain communication

**FIGURE 1:**
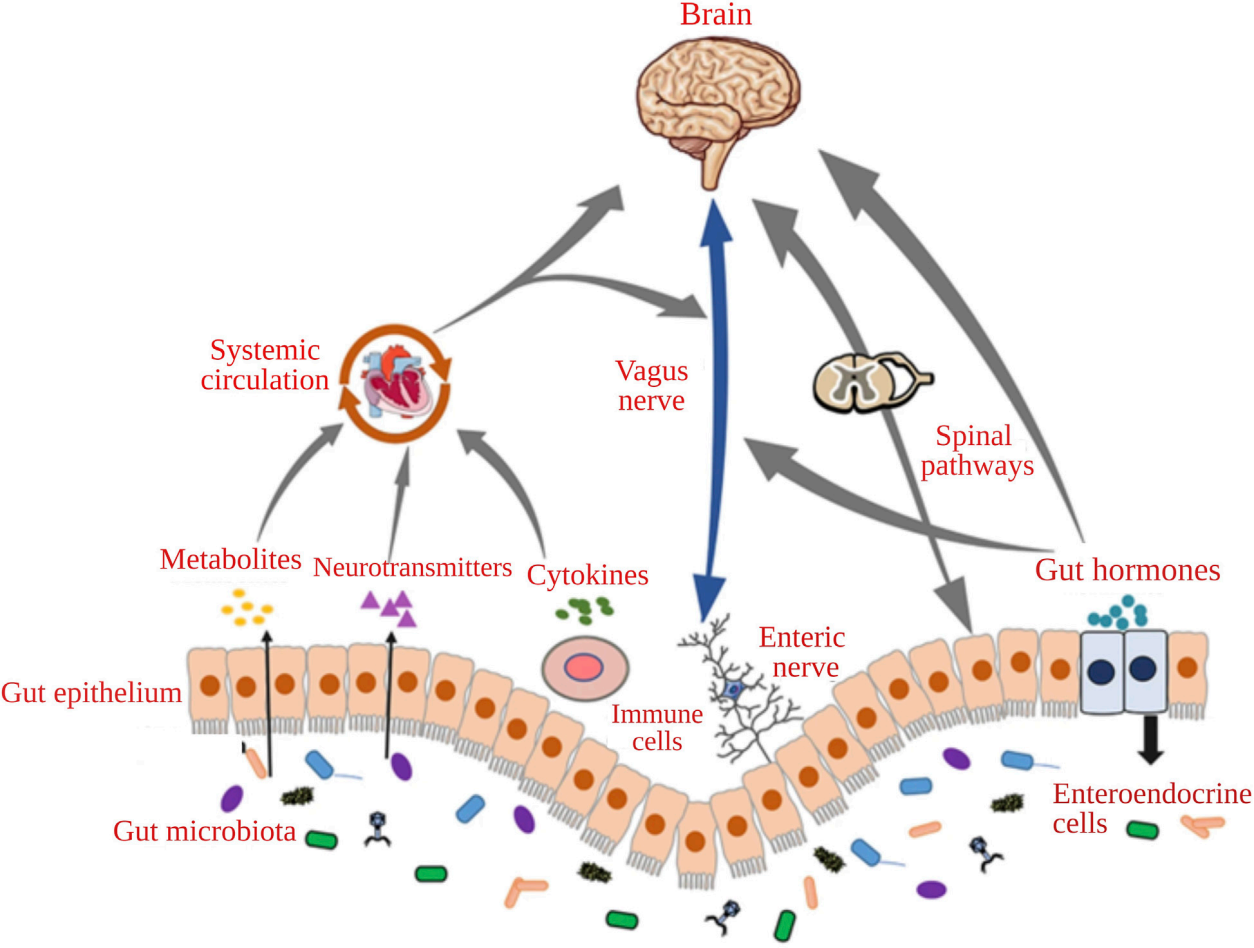
The communication pathways of the microbiota-gut-brain axis (Adapted with permission from Lin et al., 2020).

The gut and brain communicate through a complex network which includes various pathways. The neurological pathway includes the enteric nervous system (ENS), vagus nerves (VN), and gastrointestinal neurotransmitters. Modulation of afferent sensory nerves produces biologically active catecholamines and local neurotransmitters such as histamine. GM may impact the availability of nutrients, resulting in the alteration of peptide release from enteroendocrine cells and affecting the GBA. The dysbiosis of GM may cause inflammation and release of cytokines, which influence the GBA. Bacterial metabolites may affect the humoral system, cross from the BBB, and regulate microglia for cognitive development (Appleton, 2018; Gwak and Chang, 2021). The communication pathways of the microbiota-GBA have been represented in Figure 1 (Lin et al., 2020).

### 2.1 GM and GBA in cognition and neurological diseases

Studies have demonstrated that gut bacteria can produce neurotransmitters and other signaling chemicals that affect brain function and development (Morais et al., 2020; Chen et al., 2021). Recent studies detailed the associations between GM, mental health, and psychological diseases and disorders including anxiety and depression (Kumar et al., 2023). Genetics, nutrition, environment, and early life experiences play a role in mental health and development via GM. However, to completely understand the influence of GBA on human health and wellbeing, further studies are required (Kelsey et al., 2019). According to a meta-analysis, infants born via cesarean section had a slightly higher chance of developing attention deficit hyperactivity disorder (ADHD) and autism spectrum disorder (ASD) than those born vaginally (Curran et al., 2015). A study revealed that children with ASD specifically exhibit different patterns of bacterial classes, for example, ASD children have an abundance of toxin-producing bacteria, like *Clostridia* (Kelsey et al., 2019).

The association of GM with the cognitive function of the infant has been reported. Three different microbial clusters have been identified, based on the variations in the prevalence of three important bacterial species, *Faecalibacterium, Bacterioides*, and *Ruminococcaceae*. The study showed the differences between the three groups in the Early Learning Composite Score (measures an infant’s overall performance in activities involving cognitive and motor development). The newborns with a comparatively high abundance of *Bacteroides* had the greatest scores. In contrast, newborns with a reasonably high abundance of *Faecalibacterium* had the lowest cognitive and motor development scores. Also, the research findings revealed structural variations in the brain; the newborns with a substantial abundance of *Bacteroides* showing a bigger right superior occipital gyrus at age one compared to infants in the other two groups (Carlson et al., 2018). Kelsey et al. reported that gut microbial diversity at first year of life reflected cognitive development at second year of age. So, the result was predicted that the microbiome affects the functions of cognitive development differently during infancy than in later stages in the developing phases of life (Kelsey et al., 2019).

The microbiome’s initial formation and the nervous system’s growth and maturation co-occur from the very earliest stages of fetal development. Recent studies reveal the role of microbiomes in brain development and neurological diseases and disorders (Dash et al., 2022; Nandwana et al., 2022). Any issue impairing brain function might also potentially result in several neurodevelopmental disorders (NDDs) (Dash et al., 2022; Wang et al., 2022). Several NDDs have been reported, including intellectual development disorder, ADHD, ASD, specific learning disabilities like dyslexia or dyscalculia, conduct disorders, motor disorders (tic disorders, Tourette’s syndrome), and a congenital disorder-cerebral palsy (Antolini and Colizzi, 2023). The people with NDD struggle to operate in social, intellectual, professional, and personal spheres of life. Impaired brain activities affect emotions, self-control, learning capacity, memory, intelligence quotient, and social skills. The first sign and behavioral difficulties may appear in early infancy, but the entire spectrum of NDDs becomes visible when the child grows. The spectrum of developmental impairments frequently persists throughout the person’s lifetime (Thapar et al., 2017). The symptoms of NDDs might be alleviated by interventions that target GM (Vendrik et al., 2020).

A linkage was found between the brain’s functions or dysfunction and the host’s GM (Iliodromiti et al., 2023). A meta-analysis study compared GM of kids with and without ASD. The gut of children with ASD has significantly higher concentrations of *Bacteroides, Parabacteroides*, and *Clostridium* species, while *Bifidobacterium* species are lower. This dysbiosis could contribute to the development of ASD (Iglesias-Vázquez et al., 2020). Also, another study showed that autistic kids had high concentrations of specific bacteria such as *Clostridium, Desulfovibrio, Sutterella*, and *Lactobacillus*. Still, the results are inconsistent (Bezawada et al., 2020), indicating that further studies are needed to know whether the altered microbiome is the cause or consequence of the condition.

Bundgaard-Nielsen et al. reported that the people with ADHD and ASD share GM sign (both alpha and beta diversity), which was different from the control. A high abundance of *Eggerthella*, *Hungatelle* and *Ruminococcus gnavus*, and a lower relative abundance of *Coprobacter* and *Howardella* were found among the ADHD and ASD subjects (Bundgaard-Nielsen et al., 2023). Meta-analysis reported no significant differences in ADHD patients, in terms of their alpha diversity indexes. Especially, genus *Blautia* abundance was higher in ADHD subjects than controls (Wang et al., 2022). The meta-analysis showed that only heterogenic data is available on the GM of ADHD patients. So, further studies are required using more study subjects, which may explain the relationship between microbial dysbiosis and ADHD.

## 3 Role of probiotics in gut health

Probiotics are living, non-pathogenic bacteria and yeast that are beneficial for the human body when administrated and improve and promote microbial balance, particularly in the digestive system (Martín and Langella, 2019). They primarily comprise *Lactobacillus* and *Bifidobacterium* species or *Saccharomyces boulardii* (Mishra and Acharya, 2021). The probiotic strains are involved in several physiological events, including reducing the pH of the intestine, cell-to-cell signaling, lower and preventing the colonization of pathogenic microbes and regulating the immune response of the host (Iacob et al., 2019). A separate probiotic group is called “psychobiotics”, which could improve psychological and mental health and influence mood, anxiety, focus, memory, and cognition (Sharma and Kaur, 2020; Thangaleela et al., 2022).

The collection of microbes, their genomes, and their products that inhabit the human intestine is referred to as the gut “microbiome” as opposed to the microbes themselves, which are referred to as “microbiota” (De Vos et al., 2022). The most common strain of GM belongs to the phyla like *Bacteroides, Proteobacteria,* and *Actinobacteria* while the most prevalent genera are *Streptococcus, Pseudomonas, Bacteroides, Fusobacteria, Clostridium,* and *Lactobacillus* (Iliodromiti et al., 2023). The gut bacteria associated with the host chronic inflammation and defense system (Ferreira et al., 2014), preserve the underlying framework of the mucosal barrier (Paone and Cani, 2020), and aid in the host’s metabolism (Lindsay et al., 2020). GM is associated with the production of several gastrointestinal hormones, short-chain fatty acids and vitamins, and medication uptake (Mendoza-León et al., 2023). It is known that the disruption in healthy GM results in inflammation (Huang et al., 2019). Additionally, it has been claimed that inflammatory states are linked to diseases like depression and anxiety. The GBA is thought to be responsible for this phenomenon (Kumar et al., 2023).

## 4 Probiotics, cognitive development, and neurological diseases and disorders

GM may influence various brain growth and operations elements, such as microglia and astrocyte polarisation, maturation, and control of neurotransmission, neurogenesis, and myelination (Ghezzi et al., 2021). Probiotics could influence the composition of GM and restore the gut ecosystem and be used as a potential approach for preventing and treating cognitive deficits (Sivamaruthi et al., 2019; Thangaleela et al., 2022). For example, the supplementation of a probiotic mixture (*Lactobacillus acidophilus*, *L. rhamnosus* and *Bifidobacteria longum*) for 3 months improved the abundance of *Bifidobacteria* and *Lactobacilli* levels and symptoms of autism assessed by the Autism Treatment Evaluation Checklist (Shaaban et al., 2018).

Probiotics maintain a healthy environment in the intestine and reduce the various risk factors (Varela-Trinidad et al., 2022). Ishii et al. reported the facilitation of hippocampus learning and memory in mice model of Parkinson’s disease after administering *Bifidobacterium breve (B. breve)* strain A1. *B. breve* A1 supplementation recovered the transcriptional level expression of synaptophysin (SYP) and postsynaptic density protein-95 (PSD95) synaptic protein, which is involved in synaptic formation and stability (Ishii et al., 2021). Likely, the supplementation of a probiotic mixture containing *B. bifidum, B. longum, L. rhamnosus, L. rhamnosus* GG, *L. plantarum* LP28, and *Lactococcus lactis* subsp. *lactis* has improved the motor neuron function in mice models of Parkinson’s disease. Probiotic interventions could provide neuroprotection and reduce the exerts, improving dopaminergic neuronal degeneration (Hsieh et al., 2020). *B. animalis* subsp. *Lactis* and arginine administration improved cognitive flexibility in C57BL/6 mice, and the study proposed that maintaining a controlled intestinal environment with functional foods like probiotics could improve cognitive flexibility (Ikuta et al., 2023).

The administration of *L. plantarum* and *B. bifidum* in Wistar rats for 8 weeks increased the concentration of choline acetyltransferase and brain-derived neurotrophic factor in the hippocampus (Ağagündüz et al., 2022). *L. plantarum* DP189 strain helps in the regulation of the P13K/AKE/GSK-3β pathway in Alzheimer’s disease mice, resulting in the improvement of dysbiosis and prevented *tau* hyperphosphorylation (Song et al., 2022). It has been reported that *L. plantarum* PS128 aids in regulating glycogen synthase kinase 3β activity in streptozotocin-accelerated cognitive dysfunction mice (Huang et al., 2021).

There were several probiotic strains like *B. fragilis, Prevotella histicola, Lactobacillus* sp., *B. animalis*, *L. plantarum*, *L. paracasei* administered to the animal model, which showed the essential impact on the level of anti-inflammatory markers and delay the onset of multiple sclerosis. There would be enhancement in the level of Treg’s (CD4^+^ CD25^+^ Fox P3^+^) and regulate the balance of Th1/Th17 and Th2 cytokines by probiotics in multiple sclerosis (Dziedzic and Saluk, 2022).

## 5 Mechanisms underlying probiotic effects on the gut-brain axis

Gut microbial disruption can negatively impact mental health. Therefore, restoration of gut microbiota could be used as an intervention to improve mental health. Modifying the GM could affect the functioning of the hippocampus. Bacterial toxins (Lipopolysaccharides; typical consequences of dysbiosis) and amyloid beta (Aβ) may interact with certain pathways like the vagus nerve pathway, the immune pathway related to the cytokines, and the systemic pathway, which is related to hormones and neurotransmitters to enhance the permeability of blood-brain barrier, mucosal-intestine barrier and finally results in the malfunctioning of the hippocampus (Tang et al., 2021).

Probiotics play a crucial role in reducing oxidative stress by producing various antioxidant enzymes (catalase and superoxide dismutase), antioxidants (butyrate, folate, and glutathione), and chelating metal ions. Probiotics may prevent immune actions like inflammatory responses by inhibiting TLR activation (Dobielska et al., 2022). The reduced inflammatory state could enhance the blood-brain barrier integrity and improve neurological functions (Wang et al., 2023). In addition to probiotic’s antioxidant and anti-inflammatory properties, they may improve cognitive function in depression by reducing hypothalamic-pituitary-adrenal (HPA)-axis dysfunction, and by increasing monoamine levels and neuroplasticity (Dobielska et al., 2022).

Studies have demonstrated that probiotics are associated with increased expression of BDNF and may be responsible for better cognitive performance (Huang et al., 2019). Prolonged supplementation of probiotics increases the concentration of tryptophan in the peripheral system and improves mental health. Probiotics exert their antidepressant effects by upregulating enzymes involved in serotonin synthesis (Lukić et al., 2022). Probiotics can change 5-hydroxytryptamine receptors, dopamine, and protein c-Fos levels; they may be responsible for modulation in the biochemistry of the CNS (Wang et al., 2016). Probiotics (*Rouxiella badensis* subsp. *acadiensis*) also increase the expression of certain mRNA, serotonin-1A (5-HT1A), and serotonin (5-HT)-2C receptors in the hippocampus (Yahfoufi et al., 2021), which could improve cognition.

Probiotics could modify the levels of neurotransmitters and neuromodulators, including serotonin, gamma-aminobutyric acid, acetylcholine, norepinephrine, N-acetyl aspartate, dopamine, and glutamate, which regulates the brain’s activity via metabolic pathways (Chudzik et al., 2021; Dicks, 2022). Though the previous studies provide established knowledge of the possible mechanisms of probiotics-mediated cognitive improvement, further studies are needed to improve the probiotic-based treatment opportunities for cognitive declines.

## 6 Clinical trials and observational studies

Both clinical trials and observational studies are crucial for assessing the effectiveness of probiotics in cognitive health. During aging, the chances of dementia and changes in their behavior also increase. Several studies reported the impact of supplementation probiotics on cognition and GM (Table 1). The supplementation of *Lactiplantibacillus plantarum* OLL 2712 for 12 weeks reduces inflammation by lowering the abundance of certain genera such as *Oscillibacter, Monoglobus*, and *Lachnoclostridium* in elderly adults (aged >65 years). The OLL2712 supplementation improved visual and composite memory (Sakurai et al., 2022). Tang et al. reported that probiotics (Lactobacilli and Bifidobacteria) act as neuroprotective agents in cognitively impaired elderly individuals and AD patients (Tang et al., 2021). Patients with Parkinson’s disease was supplemented with probiotics containing *L. casei* Shirota (6.5 × 10^9^ colony forming units) daily for 5 weeks, which increases the bowel opening and reduces constipation, bloating, and abdominal pain. This study suggested that probiotics can effectively alleviate constipation symptoms in Parkinsons patients (Cassani et al., 2011).

**TABLE 1:** The representative studies on the influences of probiotic intervention on cognitive impairment.

Study type	Subjects	Interventions, dose, and duration	Findings	References
RDB-PCT	Elderly patients following non-cardiac surgery (*n* = 120)	Capsule containing *L. acidophilus, B. longum,* and *Enterococcus faecalis* (≥10^7^ CFU of each strain/capsule); Two capsules/day; During their hospital stay	Reduced the plasma IL-6 and cortisol levels. Probiotic supplementation could relieve post-operative cognitive impairment after non-cardiac surgery in elderly patients	Wang et al. (2021)
RDB-PCT	Healthy adults with mild Cognitive Impairment (Age 50–79 years) (*n* = 80)	*B. breve* MCC1274 (1 × 10^10^ CFU/capsule); Two capsules/day; For 16 weeks	Slow down the mild cognitive impairment symptoms. Improved the anti-inflammatory system	Bernier et al. (2021)
RDB-PCT	Elderly with memory impairment and 3rd repletion value of WLMIR. (*n* = 93)	*Limosilactobacillus fermentum* A2.8 (10^7^ or 10^8^ CFU); For 12 weeks	10^7^ CFU supplemented group showed improvement in memory and visuospatial function. 10^7^ CFU supplemented group showed improvement in memory, learning, and verbal fluency	Handajani et al. (2022)
RDB-PCT	Patients undergoing hip or knee arthroplasty. (Age ≥60 years) (*n* = 106)	*L. acidophilus, B. longum* and *Enterococcus faecalis* (>10^7^ CFU each strain); Four capsules, twice/day; During hospital stay	Improved verbal memory domain. Aid in preventing perioperative development of POCD.	Hu et al. (2023)
RDB-PCT	Healthy elderly subjects (Age ≥65 years) (*n* = 63)	*B. bifidum* BGN4 and *B. longum* BORI (1 × 10^9^ CFU each strain); Four capsules/day; For 12 weeks	Improved the mental flexibility test and stress score. Reduced stress and inflammation-causing gut bacteria	Kim et al. (2021)
RCT	Middle aged (Age 52–59 years) and older adult (Age 60–75 years) with mild cognitive impairment (*n* = 169)	*L. rhamnosus* GG (10^9^ CFU) and Prebiotic inulin from chicory root extract (200 mg)/capsule. Two capsules/day; For 12 weeks	Reduced the abundances of *Prevotella* and *Dehalobacterium.* Improved the cognitive score	Aljumaah et al. (2022)
RDB-PCT	Older adults with mild cognitive impairment (Age 65–88 years) (*n* = 130)	*B. breve* MCC1274 (2 × 10^9^ CFU); For 24 weeks	ADAS’ subscales “orientation in time” and “writing” were significantly improved. Suppressed brain atrophy progression	Asaoka et al. (2022)
RDB-PCT	Healthy older adults without cognitive impairment (Age 60–75 years) (*n* = 60)	*B. longum* BB68S (5 × 10^10^ CFU/sachet); One sachet/day; For 8 weeks	Increased the relative abundances of *Cellulosilyticum, Dorea, Lachnospira*, and *Bifidobacterium*. Decreased the relative abundances of *Porphyromonas, Bilophila, Parabacteroides, Tyzzerella, Collinsella, Epulopiscium, Granulicatella,* and *unclassified_c_Negativicutes*. RBANS score was significantly improved	Shi et al. (2022)
RCT	Older adults with mild cognitive impairment (Age >60 years) (*n* = 42)	*Lactococcus lactis* BioF-224, *Lactococcus lactis* LY-66, *B. lactis* CP-9, *B. animalis* BB-115, *B. infantis* BLI-02, *B. lactis* HNO19, *L. plantarum* CN 2018, *L. plantarum* BioF-228, *L. rhamnosus* Bv-77, *L. rhamnosus* HNO01, *L. johnsonii* MH-68, *L. paracasei* MP137, *L. paracasei* GL-156, *L. salivarius* AP-32, *L. acidophilus* TYCA06, *L. casei* CS-773, *L. reuteri* TSR332, *L. fermentum* TSF331 (>2 × 10^10^ CFU/g); 2g/day; For 12 weeks	The relative abundances of *Haemophilus, Pantoea, Erysipelotrichaceae, Anaerostipes, Ruminococcus, Prevotellaceae, Lachnospiraceae, Muribaculaceae, Coprococcus*, and *Blautia* were increased. Cognitive function (based on the MMSE and MCA scores) and sleep quality were improved	Fei et al. (2023)
RCT	Healthy adult females (Age 19–31 years) (*n* = 53)	*L. acidophilus* LA02 and *B. lactis* BS01 (2 × 10^9^ CFU/capsule); For 6 weeks	No significant impact on cognition in the healthy population	Czajeczny et al. (2023)
RCT	Adults with active physical activity (Average age ∼64.3 years) (*n* = 127)	*L. rhamnosus* GG	No significant improvement in cognitive function	Sanborn et al. (2022)

RDB-PCT, Randomized double-blind and placebo-controlled trial; RCT, Randomized clinical trial; CFU, Colony forming unit; WLMIR, Word List Memory Immediate Recall; POCD, Postoperative cognitive dysfunction; ADAS, Alzheimer disease assessment scale; RBANS, Repeatable Battery for the Assessment of Neuropsychological Status; MMSE, Mini-mental state examination; MCA, Montreal cognitive assessment.

Hu et al. reported that the supplementation of *Lactobacillus acidophilus*, *Bifidobacterium longum* and *Enterococcus faecalis* (>10^7^ CFU of each strain × 4; twice/day) improved the verbal memory domain of performance in elderly subjects. The intervention group had a lower incidence of decline in specific verbal memory tests like the Hopkins Verbal Learning Test-Revised. Hospital stay duration, mortality rates, inflammatory markers, and white blood cell levels did not change significantly in both groups (Hu et al., 2023). *Bifidobacterium bifidum* BGN4 and *Bifidobacterium longum* BORI intervention for 12 weeks reduced the inflammation-causing gut bacteria, increased blood brain-derived neurotrophic factor (BDNF), and reduced stress levels in healthy elders. The study revealed that probiotic intake had no significant impact on cognitive domains such as language, memory, visuo-spatial processing, or other executive functions. However, after 12 weeks of intervention, mental flexibility significantly improved in the probiotic group. Additionally, probiotics intake decreased stress levels and the abundance of specific bacterial genera, including *Eubacterium, Allisonella, Clostridiales,* and *Prevotellaceae* (Kim et al., 2021).

A meta-analysis by Zhu et al. (2021) reported that probiotics significantly improved cognitive functions, particularly in mild cognitive impairment. At the same time, some factors can influence these benefits, such as probiotic strains, dosage, duration of intervention, and disease severity. In another meta-analysis, the impact of probiotics on cognitive function in patients with AD, mild cognitive impairment, and PD was analyzed. The study suggested probiotics could improve insulin resistance, lipid metabolisms, and cognitive and gastrointestinal health (Xiang et al., 2022). Not all the randomized clinical trials showed the positive impacts of probiotics on the subject’s cognitive health (Table 1).

## 7 Safety considerations and side effects of probiotics

Though probiotics have health benefits, they have some limitations (Kothari et al., 2019; Sotoudegan et al., 2019; Congur and Dalgic, 2023). Probiotics may cause systemic infection by bacterial translocation, gastrointestinal side effects, transfer of antibiotic resistance genes, toxic metabolic effects, and immune stimulation, The bacterial translocation is responsible for sepsis, fungemia, bacteremia, endocarditis (Congur and Dalgic, 2023). Premature infants, people with weak immunity, are more susceptible to infections. Immune stimulation may occur through bacterial toxins, including lipoteichoic acid, peptidoglycan, and lipopolysaccharides. Also, the risk of immune activation depends upon the strain of microorganisms and the dose administered (Kothari et al., 2019). Patients with short bowel syndrome risk developing probiotic-induced d-lactic acidosis due to unwarranted bacterial growth in the small intestine (Rao et al., 2018). d-lactic acidosis encephalopathy has neurologic symptoms such as memory loss, delirium, ataxia, and dysarthria (Kowlgi and Chhabra, 2015). Generally, infants, critically ill patients, patients with compromised immune systems, and cancers are considered risky subjects (Sotoudegan et al., 2019).

## 8 Recommendations for safe probiotic use

Certain key safety points must be adopted to recommend probiotics for safer use. Microbiome profiling is recommended because it can help identify factors affecting how individuals respond to probiotics differently and test various theories and processes. Manufacturers of older strains who might not have used modern techniques of assessing the risk of antibiotic resistance should re-evaluate their strains to ensure compliance. Manufacturers ought to disclose each probiotic strain’s antibiogram and, if necessary, offer an empirical course of therapy. Research into animal models is encouraged to improve our knowledge of detecting possible long-term impacts of probiotics, particularly regarding next-generation strains. Companies must monitor and report adverse occurrences in compliance with regulatory regulations for foods, dietary supplements, and medications (Merenstein et al., 2023).

## 9 Conclusion

Preclinical studies have highlighted the effects of probiotics on neurotransmitters, brain structure, and cognitive function in animal models. At the same time, clinical trials have demonstrated potential human benefits, including improved cognitive outcomes and reduced anxiety and depression. However, the safety concerns and possible risks of probiotic usage must be closely monitored and addressed. The next-generation of probiotics, for example, *Akkermansia muciniphilia*, has protein Amuc-1100 and extracellular vesicles that help regulate the metabolic system and gut barrier integrity and reduce lipopolysaccharides leakage and inflammation. However, further investigations and microbiome characterization are required to understand individual reactions and optimize the therapeutic potential of probiotics in preventing and managing cognitive deficits.
